# Combined Application of Sodium Fluorescein and Neuronavigation Techniques in the Resection of Brain Gliomas

**DOI:** 10.3389/fneur.2021.747072

**Published:** 2021-12-06

**Authors:** Zhan Xue, Lu Kong, Shuyu Hao, Yu Wang, Guijun Jia, Zhen Wu, Wang Jia, Junting Zhang, Liwei Zhang

**Affiliations:** ^1^Department of Neurosurgery, Beijing Tiantan Hospital, Capital Medical University, Beijing, China; ^2^Department of Neurosurgery, Qingdao Municipal Hospital, Qingdao, China

**Keywords:** brain glioma, sodium fluorescein, fluorescein guided surgery, neuronavigation, treatment outcome

## Abstract

**Objectives:** To explore the effectiveness and safety of the combined application of sodium fluorescein and neuronavigation techniques in the resection of brain gliomas in different locations and patients of different ages.

**Methods:** Fifty clinical cases of brain gliomas treated at the Department of Neurosurgery of Beijing Tiantan Hospital were collected from March 2014 to March 2019. These cases were divided into a supratentorial group (24 cases) and a brainstem group (26 cases) based on location and an adult group (28 cases) and a pediatric group (22 cases) based on age. Fluorescein-guided surgery was performed: the adult group received 5 mg/kg sodium fluorescein before opening the dura, while the pediatric group received 2.5 mg/kg during resection. Tumor visualization was evaluated by the enhancement of yellow fluorescein and considered “satisfactory” if the illumination demarcated the tumor boundary. Additionally, the consistency between fluorescein and neuronavigation was analyzed. The Karnofsky performance score (KPS) of all patients was recorded and assessed at admission, discharge, and the 6-month follow-up.

**Results:** In the 28 adult cases, 4 were unsatisfactory, while in the 22 pediatric cases, 2 were unsatisfactory; in 7 cases, there was an inconsistency between yellow fluorescein enhancement and neuronavigation, 6 were in the supratentorial group, and 1 was in the brainstem group. Statistical analysis showed no significant differences in the satisfactory rate between the adult and pediatric groups (*P* = 0.575), whereas there were significant differences inconsistency between the supratentorial group and brainstem group (*P* = 0.031). The mean KPS at admission was between 70 and 100, which was not significantly different from that at discharge (*P* = 0.839), but the KPS at the 6-month follow-up was significantly higher than that at admission (*P* = 0.041).

**Conclusions:** The consistency between sodium fluorescein and the neuronavigation system was higher in the brainstem group than in the supratentorial group; a half dose of sodium fluorescein (2.5 mg/kg) was sufficient for pediatric patients. The combined utilization of sodium fluorescein and neuronavigation techniques may confer glioma patients the opportunity to obtain better clinical outcomes after surgery.

## Introduction

Gliomas can occur in different locations and patients of different ages. For tumors located in the brain stem or adhering to supratentorial functional regions, the purpose of surgery should be to maximize tumor resection while preserving important neurological functions. Therefore, demarcation of the tumor boundary from surrounding normal tissue is essential for the safe removal of the lesion. In recent years, many techniques made great contributions to tumor positioning and imaging, such as sodium fluorescein (FL), 5-aminolevulinic acid (5-ALA), and neuronavigation, all of which resulted in much safer and more effective resection of brain gliomas, leading to better outcome to the patients (1–3). Because of low cost, sodium fluorescein technology is wider applied in developing countries than 5-ALA. However, because of the diversity of anatomic structures in different intracranial areas, the accuracy and consistency of fluorescence and neuronavigation technology in different brain locations is still undetermined. In addition, although it has been proven that 5 mg/kg sodium fluorescein is effective for demarcating the tumor margin in adults while 2.5 mg/kg is sufficient for pediatric patients, a comparative observation is still needed between adult and pediatric cases with different doses of sodium fluorescein to clarify the effect of imaging (4, 5).

In this study, we retrospectively analyzed 50 cases of intracranial gliomas treated in Beijing Tiantan Hospital, Capital Medical University, from March 2014 to March 2019 to evaluate the effectiveness and safety of the combined application of sodium fluorescein and neuronavigation systems in different tumor locations and patients of different ages.

## Experimental Section

### Patients Selection and Grouping

In this study, we retrospectively reviewed patients diagnosed with intracranial glioma who were treated in the Department of Neurosurgery of Beijing Tiantan Hospital between March 2014 and March 2019. Inclusion criteria were as follows: (1) newly diagnosed or suspicious, untreated gliomas; (2) the lesions were adhered to supratentorial areas or located in the brainstem areas; (3) brainstem lesions should be identified as a focal exophytic tumor and remarkably enhanced on MRI; (4) Karnofsky performance score (KPS) ≥ 70. Exclusion criteria included: (1) histological diagnosis other than glioma; (2) previously treated, recurrent tumors; (3) diffuse intrinsic or unresectable brainstem gliomas without or with mild enhancement on MRI after contrast; (4) KPS <70; (5) medical conditions that prevent the surgery or use of sodium fluorescein, such as coagulopathy, renal and hepatic dysfunction.

According to the inclusion and exclusion criteria, we identified 50 patients with gliomas (27 men and 23 women), such as 16 glioblastomas, 21 anaplastic astrocytomas, 8 oligodendrogliomas, and 5 pilocytic astrocytomas. All the patients were divided into a supratentorial group (24 cases) and a brainstem group (26 cases) based on tumor location, and an adult group (28 cases, mean age 50.5 years, range 21–64 years) and a pediatric group (22 cases, mean age 9.1 years, range 2–14 years) based on age.

### Clinical and Radiological Evaluation

**Figure 1:**
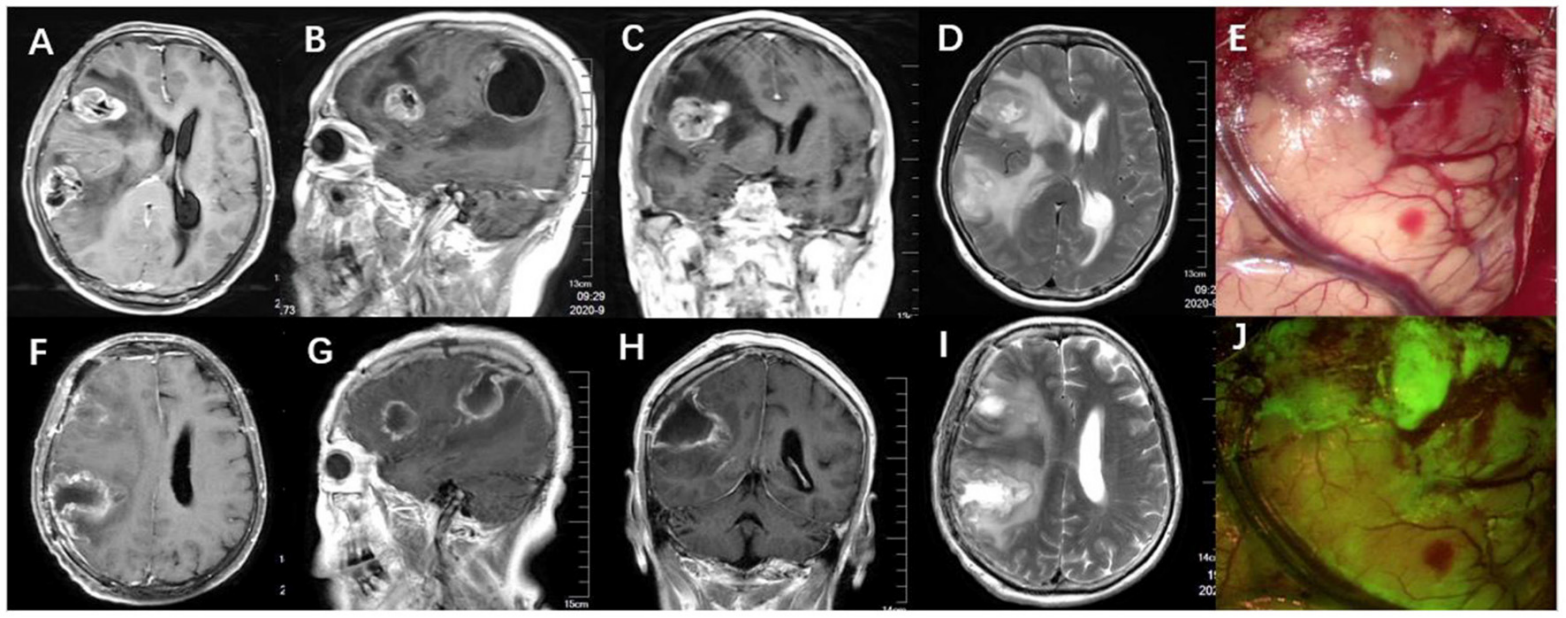
**(A–D)** Preoperative MRI scan (axial, coronal, sagittal T1-weighted with gadolinium, and T2-weighted) showed adult multifocal glioblastoma. **(F–I)** Postoperative MRI showed complete tumor removal and relief of peritumoral edema. **(E,J)** During the surgery, the tumor tissue was stained yellowish green under the fluorescein mode of the microscope and was demarcated from normal brain tissue.

**Figure 2:**
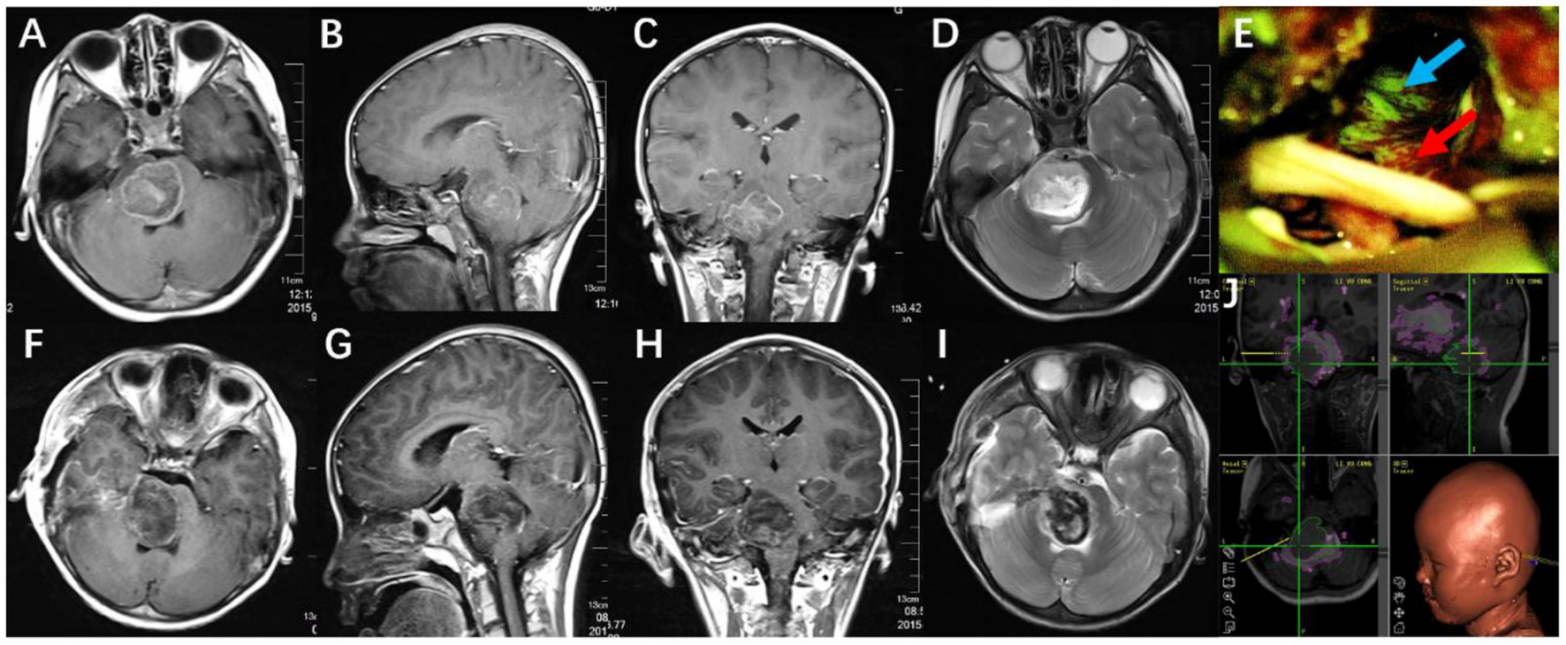
**(A–D)** Preoperative MRI scan (axial, coronal, sagittal T1-weighted with gadolinium, and T2-weighted) showed a pediatric anaplastic astrocytoma in the right pons. **(F–I)** Postoperative MRI showed subtotal tumor resection. **(E)** Tumor was visualized under the YELLOW 560 nm filter (blue arrow showed tumor, red arrow showed brain tissue). **(J)** The surgical plan was made with the assistance of neuronavigation, revealing the relationship between the tumor, white matter tracts, and brainstem nuclei.

Clinical data at admission, discharge, and 3 months of follow-up were recorded and analyzed. For the supratentorial group, eight tumors were in the frontal lobe, six in the temporal lobe, seven in the thalamus, and three in the multiple lobes, with the main presenting symptoms being headache, movement disorder, and epilepsy (Figure 1). In the brainstem group, five tumors were in the midbrain, 16 in the pontine, and five in the medulla oblongata, and the main presenting symptoms were paralysis, diplopia, and dysphagia (Figure 2). Pre- and post-contrast MRI was performed no earlier than 5 days before the surgery and no later than 3 days after the surgery. Diffusion tensor imaging was merged into the neuronavigation system (StealthStation, Medtronic, Minneapolis, USA) if the tumor adhered to or infiltrated critical areas.

### Surgical Protocol

After general anesthesia, 0.05 ml of 10% sodium fluorescein was used to perform a sensitivity test. The intraoperative use of sodium fluorescein was allowed if the test was negative. Before opening the dura, a dose of 5 mg/kg for the adult group and 2.5 mg/kg for the pediatric group was injected through the central vein. Microsurgery was performed under a PENTERO 900 microscope (Carl Zeiss, Oberkochen, Germany) equipped with a YELLOW 560 nm filter, which allowed for switching the illumination between fluorescence and white light. The neuronavigation system (Medtronic, Minneapolis, USA) was routinely used for approach planning, tumor localization, and margin demarcation. Electrophysiological monitoring was used for the tumors near or invading the functional areas. The surgical procedures were performed by the same group of neurosurgeons, and the chief surgeon was responsible for judging the effectiveness of sodium fluorescein as “satisfactory” or “unsatisfactory.” “Satisfactory” was defined as the tumor being visualized as a yellow color by sodium fluorescein and clearly different from the surrounding normal tissue, while “unsatisfactory” was considered to be a mild enhancement, vague visualization, or blurred tumor-brain interface. The tumor was removed with standard microsurgical techniques. At the end of resection, the consistency between the fluorescein guide and the neuronavigation guide was carefully compared by detecting the tumor margin with the probe under the fluorescein mode of the microscope. A “consistency” was determined if the distance of the tumor margin measured by the two techniques was <2 mm, while 2 mm or more of a brain shift was considered an “inconsistency.” All patients were followed up in the neurological intensive care unit after surgery and were transferred to the regular ward as soon as their condition became relatively stable.

### Extent of Resection

Gross total resection (GTR) was defined as no residual tumors seen on postoperative MRI, near-total resection (NTR) was defined as <5% of the tumor volume compared with the preoperative MRI, subtotal resection was defined as 5–10% residual tumor, and partial resection was defined as more than 10% of residual tumor. Tumor volume (V) was calculated by the formula V = A × B × C/2, which was determined by measuring the maximal length of axial (A), sagittal (B), and coronal (C) scans of preoperative and postoperative MRI by a vendor-provided software settled in the medical system.

### Ethical Approval

The study was conducted in accordance with the *Declaration of Helsinki* and was approved by the Ethics Committee of Beijing Tiantan Hospital (No. KY 2019-115-01). Written informed consent was obtained from each patient (or the legal guardian of pediatric patients) before enrollment in the study.

## Results

### Extent of Resection

All tumors showed enhancement on preoperative MRI, so the extent of resection (EOR) could be confirmed by comparing pre and postoperative MRI. In a total of 50 patients, GTR was achieved in 41 patients, constituting 82% of the cohort. Residual tumors (all defined as NTR) were detected in nine cases, mostly due to the remarkable adhesion or invasion of the tumors to the functional cerebral areas or brainstem nuclei.

### Intraoperative Fluorescence and Navigation Characteristics

During the 50 tumor resection surgeries, 44 of the tumors were enhanced vividly by sodium fluorescein and stained yellowish-green under the fluorescein mode of the microscope. Twenty-four tumors from the adult group and 20 from the pediatric group were identified as “satisfactory” by the neurosurgeons. Two “unsatisfactory” cases from the pediatric group were pilocytic astrocytomas, while four from the adult group were oligodendrogliomas. For the cases that identified “satisfactory,” sodium fluorescein made the tumors recognizable enough to help the surgeons dissociate the lesion from the non-fluorescent surrounding tissue. No technical difficulties were found when switching the illumination between white light and fluorescence with one button on the grip. When comparing the consistency between fluorescein and neuronavigation, we found that the tumor margin distances measured independently by the two methods were not consistent in seven cases: six cases were from the supratentorial group and one from the brainstem group (Figure 2). Statistical analysis showed no significant differences between the adult group and pediatric group on the satisfactory visualization rate by fluorescence (*P* = 0.575), but significant differences were observed between the supratentorial group and brainstem group on consistency between fluorescein and neuronavigation (*P* = 0.031) (Tables 1, 2).

**Table 1:** Visualization effect by fluorescence.

**Group**	**Division**	**Satisfactory**		**Satisfactory rate**	***P***
		**Yes**	**No**		
Age	Pediatric	20	2	90.91%	0.575
	Adult	24	4	85.71%	
Location	Supratentorial	20	4	83.33%	0.329
	Brainstem	24	2	92.31%	
Sex	Male	24	3	88.89%	0.834
	Female	20	3	86.96%	

**Table 2:** Consistency between fluorescein and neuronavigation.

**Group**	**Division**	**Consistency**		***P***
		**Consistent**	**Inconsistent**	
Age	Pediatric	21	1	0.088
	Adult	22	6	
Location	Supratentorial	18	6	0.031
	Brainstem	25	1	
Sex	Male	22	5	0.318
	Female	21	2	

### Follow Up

The statistical analysis showed no significant differences between the patients' KPS at discharge and admission (*P* = 0.839), but the KPS of the patients at the 6-month follow-up was significantly higher than that at admission (*P* = 0.041) (Table 3).

**Table 3:** Karnofsky performance score of the patients.

**Observation time**	**KPS**			
	**70**	**80**	**90**	**100**
Admission	10	15	16	9
Discharge	9	17	17	7
6 months follow up	1	17	20	12

## Discussion

Brain glioma is the most common malignant brain tumor occurring in both adult and pediatric patients. Glioma can grow in various locations in the brain tissue. For lesions found adhering to functional cerebral regions or focally invading the brain stem near important nuclei (often named exophytic brainstem gliomas), the treatment strategy remains challenging. Although there has been significant progress in radiotherapy, chemotherapy, and even immunotherapy, surgery is still considered the preferred treatment for cerebral or exophytic brainstem gliomas if the patients have no surgical contraindication (6–8). With the popularization of advanced adjuvant techniques, such as neuronavigation, neurophysiological monitoring, and intraoperative MRI, the safety and effectiveness of surgeries have greatly improved. However, for most neurosurgeons, “having a clear visual field” during tumor resection is one of the most essential principles, resulting in a higher resection rate, less risk of intraoperative hemorrhage, and better neurological status after surgery. Sodium fluorescein, indocyanine green (ICG), and 5-aminolevulinic acid (5-ALA) are the most often used reagents for intraoperative visualization during tumor removal. In 1998, 5-ALA was first reported in the resection of gliomas by Stummer et al. (9), which have significantly proven to improve the EOR of high-grade gliomas (2). Although 5-ALA has been approved by the FDA for glioma surgery in 2017 (10), in some developing countries like China, it is expensive in reagent purchasing and microscope upgrading. In comparison to ICG, fluorescein-guided surgery has obvious advantages, such as continued microsurgical work in filter mode, low cost, and easy preparation before surgery (11). The fluorescein technique was first introduced in 1947 for clinical use in brain tumor resection (12), and after the YELLOW 560 nm filter mode for microscopes was developed in 2012, the application of fluorescein-guided surgery blossomed. At present, the intraoperative fluorescein visualization technique has been applied in the resection of high-grade gliomas (1, 13), cerebral metastases (14, 15), primary central nervous system lymphomas (16), and gangliogliomas (11). Until February 2018, at least 1,099 patients with brain tumors underwent surgery with the assistance of fluorescein, mostly after 2012 (17).

The basic theory of fluorescence visualization for intracranial malignant tumors is that fluorescein can penetrate the incomplete blood–brain barrier. In 1982, Murray reported the application of sodium fluorescein for the resection of malignant brain tumors, with 84.7% sensitivity and 94.7% specificity for the fluorescein guidance (18). Shinoda and Koc also reported studies utilizing the sodium fluorescein in the glioblastoma surgery and found it “simple and safe” during the tumor resection (19, 20). In 2013, Acerbi first reported that fluorescein-guided surgery for grade IV gliomas under a specific microscope equipped with a filter (YELLOW 560 nm) and concluded that advances in microscope technology could help better visualize the tumor (21). In 2017, the FLUOGLIO study confirmed that the fluorescein-guided technique with a dedicated filter on the surgical microscope is safe and enables a high percentage of contrast-enhancing tumors in patients with high-grade gliomas (22). Bowden analyzed the relationship between areas of fluorescence and pathology and showed that samples from areas of fluorescence demonstrated greater total cell density and higher Ki-67 (23). However, in the same study, they also found 11 cases of non-tumor tissue appeared illumination by the fluorescein. In our study, by utilizing sodium fluorescein, 44 cases (88%) were considered “satisfactory” in demarcating the tumor boundary, with a relatively high resection rate. The “unsatisfactory” cases in our study were pilocytic astrocytomas and oligodendrogliomas, which may verify the theory that the fluorescein cannot penetrate sufficiently through the blood–brain barrier in low-grade gliomas, which is in line with the published studies (1, 13, 21, 22). Also we found that the fluorescence characteristics were irrelevant to the location of the lesions, which may indicate that fluorescein is useful in various areas of brain tissue; luminance may only be correlated with malignancy.

Other factors that influence fluorescence visualization are the dose of sodium fluorescein and the delivery time (24). In the early stage of the study, the dose was 20 mg/kg to visualize the tumor by the naked eye and under the white light (15, 19, 20). However, some authors criticized that a high dose of sodium fluorescein may cause allergic shock (25). After using a microscope in the filter mode, the dose was further reduced to 5 mg/kg (1). In our previous report, 12 pediatric patients with exophytic brainstem glioma were given sodium fluorescein at 2.5 mg/kg and showed satisfactory fluorescence images without adverse effects (5). In this study, we found no differences between the adult group (5 mg/kg) and pediatric group (2.5 mg/kg) in the fluorescence visualization, which indicated that a half dose in children can achieve a similar degree of visualization as in the adult, which may further improve safety and reduce adverse effects. Sodium fluorescein was usually administered during anesthesia induction, incision of the scalp, opening of the dura, or exposure of the tumor (17). In our study, sodium fluorescein was administered intravenously when opening the dura, which was proven reasonable in achieving the satisfactory fluorescein images.

Neuronavigation has often been utilized during surgery for brain tumor resection since it was first developed and reported by Roberts in 1986 (26). One of the advantages of the neuronavigation to glioma surgery is that, with the help of a preoperative plan, it allows neurosurgeons to recognize nuclei and white matter fibers that are invisible under the microscope and thus may result in the better neurological outcomes. However, the primary disadvantage of neuronavigation is the “brain shift.” In the older series, the shift was reported to be more than 10 mm (3). With the updates in navigation equipment, the range of system error was reduced. In our study, we identified 2 mm or less as the system error and found more inconsistency between fluorescein and navigation in tumors located in the supratentorial areas than in the brainstem areas. This may indicate that the accuracy for neuronavigation in the supratentorial area is not reliable and requires more attention when combined with sodium fluorescein.

In our study, EOR was not compared with the cases not applied with sodium fluorescein or navigation, which is beyond the scope of this study and will be evaluated in future research. The KPS is very important in measuring the quality of life of patients. In our study, the KPS at discharge was not higher than that at admission but significantly improved at the 6-month follow-up. The reason for this may be the vulnerability of the nerve tract and nuclei, which may be temporarily impaired shortly after the operation.

## Conclusions

Our data and surgical experiences verified the feasibility and safety of sodium fluorescein in surgery for brain gliomas. For different tumor locations, the consistency between sodium fluorescein and neuronavigation techniques was higher in the brainstem group than in the supratentorial group. A half dose of sodium fluorescein (2.5 mg/kg) is adequate for the pediatric patients to obtain comparable images similar to that of adults. The combined utilization of sodium fluorescein and neuronavigation techniques may confer glioma patients with opportunities to obtain better clinical outcomes after surgery.

## Data Availability Statement

The original contributions presented in the study are included in the article/supplementary material, further inquiries can be directed to the corresponding authors.

## Ethics Statement

The studies involving human participants were reviewed and approved by Ethics Committee of Beijing Tiantan Hospital. Written informed consent to participate in this study was provided by the participants' legal guardian/next of kin. Written informed consent was obtained from the individual(s), and minor(s)' legal guardian/next of kin, for the publication of any potentially identifiable images or data included in this article.

## Author Contributions

All authors listed have made a substantial, direct, and intellectual contribution to the work and approved it for publication.

## Funding

This study was supported by grants from Beijing Hospital Authority Incubation Program (PX2019017).

## Conflict of Interest

The authors declare that the research was conducted in the absence of any commercial or financial relationships that could be construed as a potential conflict of interest.

## Publisher's Note

All claims expressed in this article are solely those of the authors and do not necessarily represent those of their affiliated organizations, or those of the publisher, the editors and the reviewers. Any product that may be evaluated in this article, or claim that may be made by its manufacturer, is not guaranteed or endorsed by the publisher.
